# *Streptococcus agalactiae* Non-Pilus, Cell Wall-Anchored Proteins: Involvement in Colonization and Pathogenesis and Potential as Vaccine Candidates

**DOI:** 10.3389/fimmu.2018.00602

**Published:** 2018-04-05

**Authors:** Giampiero Pietrocola, Carla Renata Arciola, Simonetta Rindi, Lucio Montanaro, Pietro Speziale

**Affiliations:** ^1^Unit of Biochemistry, Department of Molecular Medicine, University of Pavia, Pavia, Italy; ^2^Research Unit on Implant Infections, Rizzoli Orthopaedic Institute, Bologna, Italy; ^3^Department of Experimental, Diagnostic, and Specialty Medicine, University of Bologna, Bologna, Italy; ^4^Department of Industrial and Information Engineering, University of Pavia, Pavia, Italy

**Keywords:** group B *Streptococcus*, cell wall anchored-proteins, extracellular matrix, adherence, invasion, immune system, pathogenesis, vaccines

## Abstract

Group B *Streptococcus* (GBS) remains an important etiological agent of several infectious diseases including neonatal septicemia, pneumonia, meningitis, and orthopedic device infections. This pathogenicity is due to a variety of virulence factors expressed by *Streptococcus agalactiae*. Single virulence factors are not sufficient to provoke a streptococcal infection, which is instead promoted by the coordinated activity of several pathogenicity factors. Such determinants, mostly cell wall-associated and secreted proteins, include adhesins that mediate binding of the pathogen to host extracellular matrix/plasma ligands and cell surfaces, proteins that cooperate in the invasion of and survival within host cells and factors that neutralize phagocytosis and/or modulate the immune response. The genome-based approaches and bioinformatics tools and the extensive use of biophysical and biochemical methods and animal model studies have provided a great wealth of information on the molecular structure and function of these virulence factors. In fact, a number of new GBS surface-exposed or secreted proteins have been identified (GBS immunogenic bacterial adhesion protein, leucine-rich repeat of GBS, serine-rich repeat proteins), the three-dimensional structures of known streptococcal proteins (αC protein, C5a peptidase) have been solved and an understanding of the pathogenetic role of “old” and new determinants has been better defined in recent years. Herein, we provide an update of our current understanding of the major surface cell wall-anchored proteins from GBS, with emphasis on their biochemical and structural properties and the pathogenetic roles they may have in the onset and progression of host infection. We also focus on the antigenic profile of these compounds and discuss them as targets for therapeutic intervention.

## Introduction

*Streptococcus agalactiae* [group B *Streptococcus* (GBS)], an opportunistic Gram-positive human pathogen, is a major causative agent of pneumonia, sepsis, and meningitis in neonates and a serious cause of disease in parturient women and immunocompromised and elderly people. *S. agalactiae* is also a frequent cause of bone and joint infections (1, 2) and infections associated with orthopedic medical devices (3). On the basis of the antigenic and structural properties of capsular polysaccharide (CPS) GBS can be classified into 10 different serotypes (Ia, Ib, II, III, IV, V, VI, VII, VIII, and IX) (4, 5). Epidemiological investigations in the United States and Europe show that serotypes Ia, Ib, III, and V account for 85–90% of all clinical isolates (6, 7). GBS is found in the intestine and urogenital tracts of adult women and these women’s newborn babies may develop infection during exposure to the bacterium before birth or during the neonatal period. In the neonatal period, GBS can first be localized in the human vaginal epithelial cells, where it specifically attaches to molecules such as fibrinogen (FBG), fibronectin (Fn), and laminin (Lm), largely abundant in the extracellular matrix (ECM) and basement membranes. GBS can then infiltrate the uterine compartment of pregnant mothers where newborn aspirate GBS *in utero* or at birth (8). From there bacteria move toward the neonatal lung and gain access to the blood stream of the neonate with consequent bacteremia and sepsis syndrome. Finally, GBS invades multiple neonatal organs and penetrates the blood–brain barrier (BBB) eventually causing meningitis (8). Upon invasion and penetration into deep tissues, GBS are opsonized by antibodies and the complement system and ingested and killed by host phagocytic cells (neutrophils and macrophages) (9). To be successful, GBS, at each stage of this complex infective process, require the coordinate production and involvement of several factors. To mediate adherence to ECM and host cell surface, *S. agalactiae* expresses several surface-associated protein families including sets of cell wall-anchored (CWA) proteins and lipoproteins and a myriad of secreted proteins that can re-bind to the bacterial cell surface. Typical examples of GBS adhesins are the CWA FBG-binding protein A (FbsA) (10) and the secreted Lm-binding protein Lmb (11). Additional structures involved in adhesion are pili, long filamentous structures extending from the surface made up of the covalent polymerization of pilin subunits (12, 13). Other CWA and secreted proteins promote invasion of host epithelial cells. For example, alpha C protein (ACP), a CWA protein (14, 15), and FbsB, a secreted FBG-binding protein (16), both enhance internalization of *S. agalactiae* into epithelial cells. Translocation through placental, epithelial or endothelial barriers is facilitated by specific virulence determinants such as the pore-forming toxin known as β-hemolysin/cytolysin (17–19) and surface protein plasminogen-binding surface protein (PbsP), which captures plasminogen (PLG) (20). GBS expresses several CWA proteins such as GBS immunogenic bacterial adhesion protein (BibA) (21) and the secreted protein complement inhibitory protein (22) with functions in avoidance of the host immune defense system. Finally, an additional factor targeting the innate immune system includes an extracellular DNA nuclease named NucA which degrades the DNA matrix comprising the neutrophil extracellular traps (23). In view of the pivotal role of CWA proteins in the different steps of colonization and host infection, here we review the most recent discoveries on GBS CWA proteins and the functions they play as factors promoting adherence to ECM components and/or in the internalization process. The function of these proteins as determinants of resistance to innate immune clearance is also examined.

## The ECM

### General Properties

The ECM, a fundamental component of vertebrate tissues, is an environment consisting of all secreted molecules immobilized in the space between cells. Cells are anchored to the ECM mainly *via* integrins. The complete ensemble of ECM proteins constitutes the “matrisome.” The matrisome essentially consists of the “core matrisome” which includes components such as collagen, proteoglycans, and glycoproteins (Fn, Lm, etc.) and matrisome-associated proteins that can comprise a variety of functionally and structurally different proteins ranging from annexins and complement proteins to enzymes that collaborate to signal to the cells (ADAM metalloproteases, transglutaminases, etc.) and growth factors (chemokines, interferons, hormones). The components of the ECM and surface receptors that anchor cells to ECM determine the generation of various signaling pathways that control proliferation, survival, differentiation, and migration of the cells. In addition to being important for development of tissues and organs, structural modifications of the ECM have been associated with various pathologies such as cancer and fibrosis (24). GBS and other bacteria use CWA proteins to adhere to the ECM components, particularly Fn, collagens, and FBG. It is plausible that, similarly to other Gram-positive bacteria such as *Streptococcus pyogenes*, as soon as a more detailed functional analysis of other GBS CWA proteins comes to light, additional specific interactions of CWA proteins with other ECM components (e.g., laminin, vitronectin, thrombospondin) will be revealed. Thus, a challenge for investigators is to identify the ligands bound to these proteins and their role in GBS colonization and pathogenesis.

### ECM Components and Integrins

#### Fibronectin

**Figure 1:**
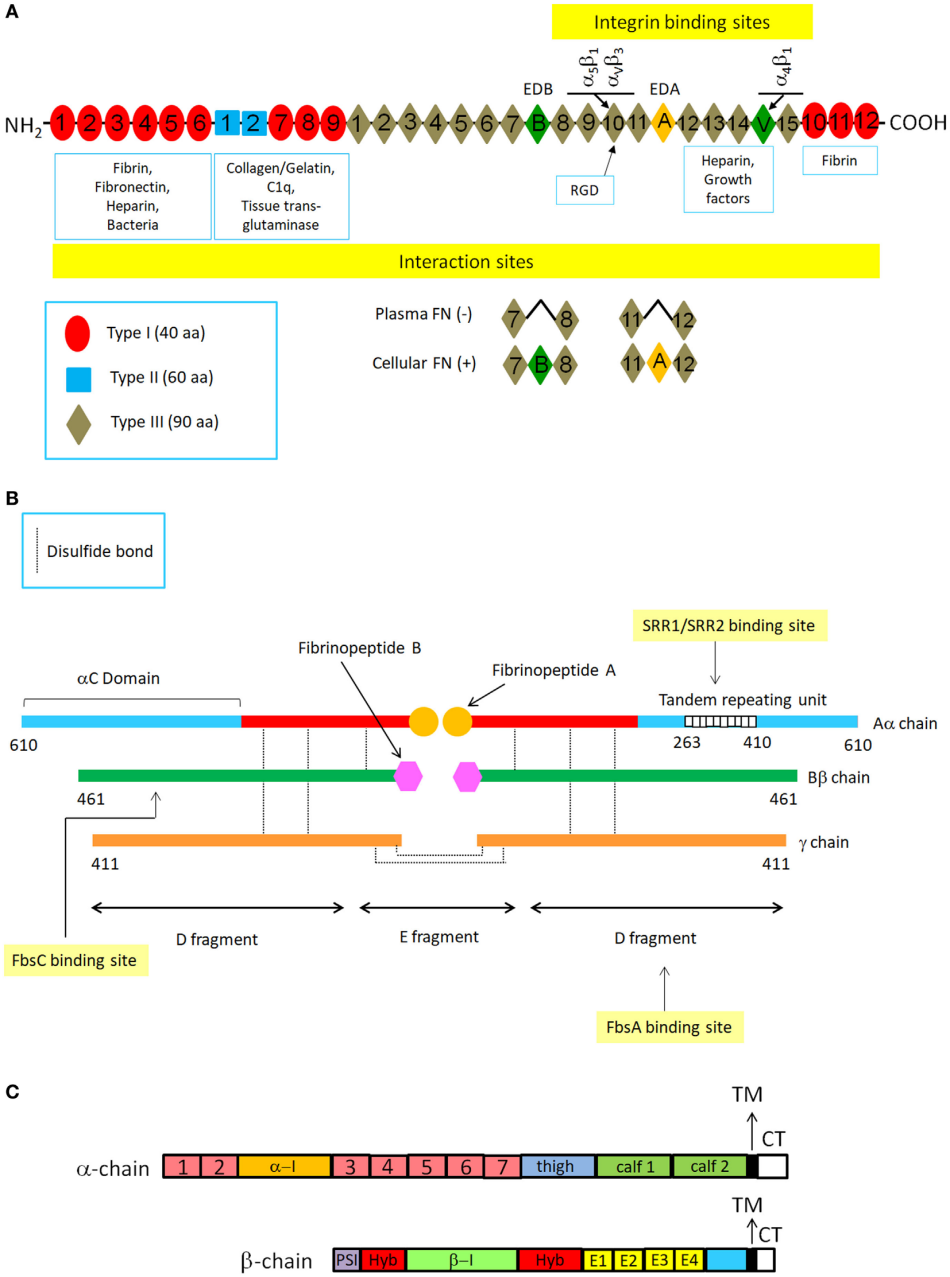
Schematic structures of typical extracellular matrix proteins fibronectin and fibrinogen and cell surface integrins. **(A)** Fibronectin (Fn) structure. The drawing shows a representation of a plasma and cellular Fn dimer and its interaction with specific ligands. Fn is a large C-terminally disulfide linked heterodimer. In each monomer there are 12 type I repeats clustered in three groups, two adjacent type II repeats and 15–17 type III repeats. Type I, II, and III repeats consist of 40, 60, and 90 amino acids, respectively. Type I and type II repeats have a specific conformation maintained by pairs of intramodule disulfide bonds, whereas the type III repeat lacks disulfide bonds. The variation in subunit size arises from alternative splicing of three segments: two type III repeats (EDA and EDB) and a third non-homologous segment known as V (variable) or IIICS. Numbering of type III homologies does not include EDA and EDB subdomains. Constitutive sequence RGD within the tenth FnIII module is indicated together with its integrin receptors. **(B)** Fibrinogen structure. FBG molecule is composed of two halves of disulfide-bridged Aα-, Bβ-, and γ-chains. Each half contains two outer globular D domains, comprising the C-terminal residues of the α-, β-, and γ−chains and connected to a central E domain by a coiled-coil segment. Fibrin formation from FBG starts following thrombin cleavage of fibrinopeptide A from Aα chains. Binding sites on FBG chains or domains for some CWA are also shown. **(C)** Integrin structure. Integrins are heterodimers composed of α and β chains, each containing around 1,000 and 750 aa, respectively. The α chain contains four/five extracellular domains: a seven bladed β-propeller, a thigh and two calf domains. Some α chains have a α-I domain inserted between the second and the third of the seven blades of the β propeller. The β chain has seven domains: a β-I domain (β-I) is inserted in a hybrid domain (hyb) located immediately after the N-terminal plexin-semaphorin integrin (PSI) domain. These domains are followed by four cysteine-rich epidermal growth factor-like motifs (E1-E4) and a β-tail domain. Both the α and β chains have single transmembrane segments (TM) and short unstructured cytoplasmic tails (CT) that vary in length. Although evidence has been obtained that several CWA proteins such as ACP interact with integrins, molecular details of these interactions have not been produced yet.

Fibronectin is a secreted, large dimeric glycoprotein composed of almost identical subunits that is found in body fluids and the ECM. Functional roles of Fn include ECM assembly, wound repair and angiogenesis (25). The subunits in the dimer are held together by disulfide bonds near their C-termini. Each subunit is made up of repeating modules of three types (I, II, and III) (Figure 1A). Alternative mRNA splicing generates monomeric subunits which are similar but not identical and that distinguish plasma from cellular isoform of Fn. Adjacent modules are organized to form discrete binding domains for a multitude of binding partners (proteins or carbohydrates). The N-terminal domain (NTD), consisting of five type I repeats, has multiple ligands such as fibrin, heparin, thrombospondin-1, and a large variety of bacterial adhesins. The NTD is also the major site for Fn self-association. Immediately close to this domain is a region composed of type I and type II modules that binds to gelatin/collagen. The large central domain, composed of 15 type III repeats, includes several binding sites finalized to establish interactions with the NTD as well as sites involved in the interaction with integrins or membrane proteoglycans such as syndecans (24, 26) (Figure 1A).

#### Fibrinogen

Fibrinogen is a soluble plasma glycoprotein synthesized by hepatocytes. It has a rod-like shape and is composed of two sets of disulfide-bridged Aα-, Bβ-, and γ-chains. The C-termini of Bβ- and γ-chains cooperate to form two D domains, while the N-terminal regions of the three chains concur to form the central E domain. The D and E domains are connected by a coiled-coil segment of the three chains. FBG is converted to fibrin after thrombin cleavage of 16-residue fibrinopeptide A from Aα-chains, thus triggering the polymerization process. The exposed end of the α chain interacts with sites in the α chain C-termini of neighboring molecules, initiating the non-covalent end-to-end and lateral assembly of the two-molecules-thick fibrin protofibrils. A second thrombin cleavage then releases a 14-residue peptide from the N-terminus of the Bβ chain and the exposed new N-terminus reinforces lateral aggregation resulting in the formation of thick fibrin fibers. After assembly of the fibrin polymer, the transglutaminase, factor XIII, stabilizes the structure by crosslinking covalently the α and β chains. In addition to its role in clotting, FBG binds to the platelet integrin, GPIIb-IIIa, contributing to the thrombus formation and interacts with other specific integrin receptors on endothelial cells and leukocytes (Figure 1B).

#### Integrins

The dominant receptors for the ECM belong to the integrin family. Integrins in mammals have eighteen α and eight β subunits and through different combinations of the α and β subunits, around 24 unique αβ heterodimeric integrins are generated. Some integrins are widely expressed, whereas others have a limited distribution in tissues where they play specific biological functions. The α- and β-subunits are composed of distinct domains joined by flexible linkers (Figure 1C). Each subunit contains a single hydrophobic transmembrane segment and a short unstructured cytoplasmic tail of 40–70 amino acids. Outside the cell membrane, the α and β chains associate to form binding regions for the ECM components. The portion of the α subunit that participates in ligand binding assumes a globular conformation and consists of seven repeats of about 60 amino acids that form a seven-bladed structure called β propeller. A significant number of the integrin α subunits have an insertion called the I-domain within the β-propeller, which plays a direct role in binding ligand. All β subunits contain an “I-like domain,” also involved in ligand binding. Both I and I-like domains contain a “metal ion-dependent adhesion site” which binds Ca^2+^ or Mg^2+^ cations which in turn stabilize the folds of the subunits or coordinate ligand binding. Most integrins interact with ECM components such as Fn and FBG through the classical integrin-binding motif Arg-Gly-Asp (RGD) (Figures 1A,C). Bacteria, including GBS, and a large number of viruses interact with integrins (27, 28). In addition, to binding extracellular ligands, integrins provide transmembrane links to a wide variety of cytoskeletal, adaptor and signaling proteins. Accordingly, the cytoplasmic domains of integrins assemble large complexes of proteins, which regulate cytoskeleton assembly and activate a number of signaling pathways within cells (24).

## The Complement System and its Regulation

**Figure 2:**
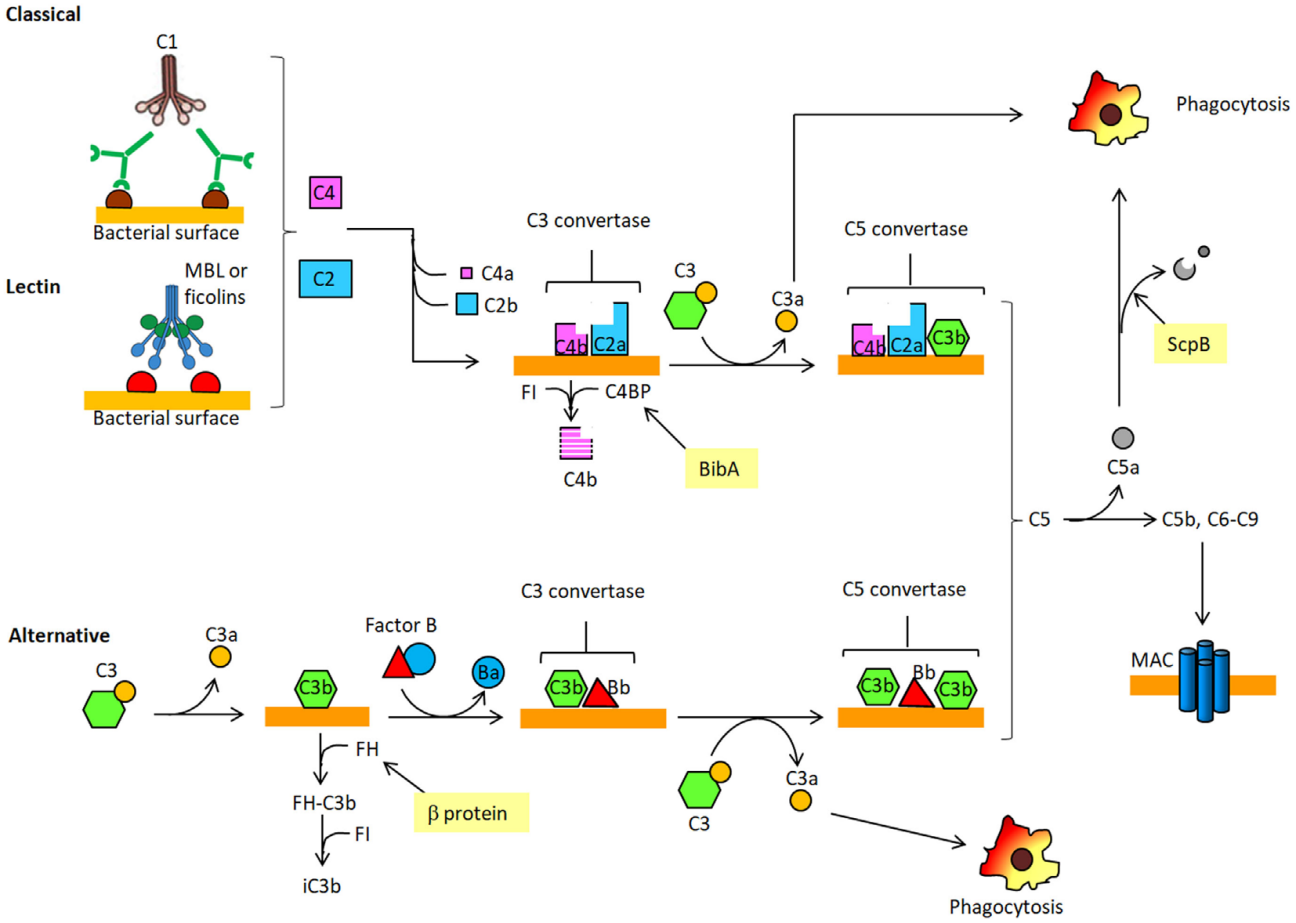
Schematic overview of the complement. The complement cascade is activated when antibody-coated bacteria are recognized by C1 complex of the classical pathway (CP). Alternatively, the complement cascade can be activated after binding of mannose-binding lectin (MBL) to sugars displayed on bacterial surface and complex so formed associates with the serine protease MASP [lectin pathway (LP)]. Both C1 and MASP cleave C4 and C2 to produce C3 convertase complex (C4bC2a) on the microorganism surface. C3 convertase cleaves C3 into C3a, an anaphylotoxin, and C3b, which binds covalently to the bacterial surface. The C3 convertase (C3bBb) of the alternative pathway (AP) is generated after complex formation between the activated product of B (Bb) and surface bound C3b. C3b also concurs to generate C5 convertases, C4bC2aCb3b and C3bBbC3b, which cleave C5 into C5a, a chemotactic peptide that attracts neutrophils to the site of infection. C5a can be inactivated by the proteolytic attach of ScpB. C5b, the other product of C5 cleavage, forms a complex with C6–9 proteins to generate the membrane attack complex (MAC). C4BP, recruited on the bacterial surface by BibA, is a cofactor of a serine protease factor I (FI) which cleaves C4b, thus preventing the formation of the classical C3 convertase. Factor H (FH) captured by β protein on GBS surface controls the activity of the complement alternative pathway by binding C3b and facilitating its cleavage by FI.

Microorganisms have developed many ways to evade host defenses, in particular complement which is the primary humoral branch of the innate immune system. Complement comprises more than 50 circulating and membrane-associated proteins. The main function of complement is to detect and eliminate pathogens. The complement system can generate the following biological effects: (i) facilitate phagocytosis of pathogens; (ii) attract macrophages and monocytes to the site of infection; and (iii) directly kill pathogens. The complement system consists of three distinct pathways: the classical, the lectin and the alternative. The classical pathway is triggered when the multimeric collectin C1q binds to immunoglobulins bound to specific antigens on the surface of microrganisms. C1q circulates in the blood stream in complex with C1r and C1s to form C1. Such binding to immunoglobulins triggers a cascade of proteolytic events which results in the cleavage of complement protein C4 in C4b and C4a proteins. C4b can then bind covalently to bacterial surfaces and associates with C2a, another proteolytic fragment generated from C2 protein. The complex of C4b and C2a (C4bC2a), termed C3 convertase, is able to cleave C3, the most abundant complement protein in serum, into C3b and C3a. The second complement pathway or lectin pathway (LP) begins when the lectin named mannose-binding protein (MBP) binds to mannose or N-acetyl glucosamine residues on bacterial surface which then associate and activate an MBP-associated serine protease (MASP). In turn, this complex cleaves C4 and C2 and the released C4b and C2a associate to form C3 convertase, as above. C3b generated by the classical or LP binds covalently to bacteria and enables them to be phagocytosed by leukocytes. In the alternative pathway the small quantity of circulating C3b spontaneously formed by hydrolysis of an internal thioester bond within C3 binds to the surface of bacteria where it interacts with a single chain plasma protein factor B, which in turn is cleaved by the serum serine protease, called factor D, to produce Bb and Ba fragments. The association of C3b with Bb generates the C3 convertase of the alternative pathway. C3b produced by the action of the three complement pathways not only participates in the opsonization of bacteria but also concurs to the formation of the C5 convertases (C4b2a3b and C3bBb3b). The C5 convertases cleave C5 into C5a and C5b. C5a and C3a are potent chemoattractants (anaphylotoxins) for phagocytic cells. C5b, along with other complement proteins, is involved in the assembly of the membrane attack complex (MAC), which can then be incorporated into the membrane of certain pathogens to form pores and induce bacterial lysis. Activation of complement is tightly regulated through the inhibition of complement serine proteases, decay of the convertases and regulation of the MAC assembly. Typical examples of regulators of complement activation are factor H (FH), C4-binding protein (C4BP), and decay accelerating factor (Figure 2) (29).

## CWA Proteins of *S. agalactiae*

### Definition and General Properties

The genomes of *S. agalactiae* strains are predicted to encode up to 35 distinct CWA proteins (30, 31). However, the exact repertoire of CWA proteins varies from strain to strain, depending on growth conditions and the phase of growth. In general, the CWA proteins have a secretory N-terminal signal sequence that directs the translation products to the cytoplasmic membrane where it is then removed during secretion. Located at the C-terminus is a sorting signal comprising a conserved sorting motif (LPXTG, Leu-Pro-Thr-Gly) (32), a hydrophobic transmembrane segment and a positively charged cytosolic terminal tail. The LPXTG sequence is cleaved between Thr and Gly by the housekeeping transpeptidase sortase A, an enzyme that also promotes covalent anchorage of the protein to cell wall peptidoglycan (31). SrtA is also involved in covalent anchoring of GBS pili to the cell wall (33). The central moiety of CWA proteins includes domains that are directly involved in one or more functional activities, among these, the ability to mediate recognition of and binding to ECM components and recruitment of specific components/modulators of the complement system. Due to the limited number of CWA proteins expressed on the cell surface, a single CWA protein can have several biological functions, a property common to CWA proteins expressed by other bacterial species (34). For example, the β protein interacts with the Fc portion of IgA, FH, and sialic-acid-recognizing immunoglobulin superfamily lectin (Siglec)-5/Siglec-14. Furthermore, many CWA proteins from GBS display functional redundancy: for example, of the cell wall anchored-proteins so far identified in GBS, at least four [FbsA, fibrinogen-binding surface protein C (FbsC), serine-rich repeat proteins (Srr) 1, and Srr2] bind to the ECM/plasma glycoprotein FBG, possibly by different mechanisms. Likewise, bacterial surface adhesin of GBS (BsaB) and streptococcal C5a peptidase B (ScpB) both interact with fibronectin and several CWA proteins [ACP, ScpB, GBS surface protein A (BspA)] bind to and mediate invasion of epithelial cells. This redundancy may result in a clear advantage for bacteria because if a given biochemical function attributed to a specific protein fails, the fitness of the organism does not substantially change because that loss of function is compensated by the activity expressed by other functionally similar proteins. Unlike CWA proteins examined in species such as *S. aureus* where detailed studies of the crystal structure have clarified the mechanism of ligand binding of several CWA proteins (34), structural and mechanistic aspects of interactions of CWA proteins from GBS with the appropriate ligands, except for the case of Srr proteins (35), await to be revealed. This lack of information concurs to make a classification of GBS CWA proteins difficult (Table 1).

**Table 1:** The main known CWA proteins of *S. agalactiae*.

Protein	Ligand and binding mechanism	Function	Reference
**CWA as adhesins/invasins and immunity regulators**			
Alpha C protein (ACP)	Heparin and glycosaminoglycans	Invasion of non-phagocytic cells	(36–38)

	α_1_β_1_ integrin	Internalization of non-phagocytic cells	(39)

β protein, Bac	Cα2/Cα3 interdomain in human IgA-Fc	Interference with IgA effector function	(40–44)

	Consensus repeats 8–11 and 12–14 of human Factor H	Regulation of complement activation	(45, 46)

	Human lectins Siglec-5 and Siglec-14	Modulation of innate immunity	(47, 48)

GBS immunogenic bacterial adhesion protein (BibA)	Human C4-binding protein	Regulation of complement activation	(21)

	Unknown ligand	Attachment to epithelial cells	(21)

Serine rich repeat protein 1 and 2 (Srr1 and Srr2)	Tandem repeats 6–8 of fibrinogen Aα chain; (dock, lock, and latch mechanism)	Adhesion to immobilized fibrinogen; attachment to endothelial cells	(49–51)

	C-terminal region of cytokeratin 4	Attachment to epithelial cells	(52, 53)

Leucine-rich repeat protein of GBS (LrrG)	Unknown Ligand	Attachment to epithelial cells	(54)

Group B *Streptococcus* surface protein A (BspA)	Gp340	Attachment to epithelial cells	(55)

Fibrinogen-binding surface protein A (FbsA)	Fibrinogen	Adhesion to immobilized fibrinogen; anti-phagocytic activity; aggregation of platelets	(10, 56)

	Unknown ligand	Adhesion to endothelial and epithelial cells	(57, 58)

Plasminogen-binding surface protein (PbsP)	Plasminogen	Extracellular proteolytic activity	(20)

	Unknown ligand	Transmigration across brain endothelial cells	(20)

Hypervirulent GBS adhesion (HvgA)	Unknown ligand	Attachment to endothelial and epithelial cells	(59)

Fibrinogen-binding surface protein C (FbsC)	Fibrinogen	Attachment to and invasion of epithelial cells	(60, 61)

	Unknown ligand	Biofilm formation	(60)

Bacterial surface adhesin of GBS (BsaB)	Fibronectin, laminin	Attachment to epithelial cells	(62)

	Unknown ligand	Biofilm formation	(62)

**CWA proteins with enzymatic activities**			

*Streptococcus agalactiae* pullulanase (SAP)	Pullulan, glycogen and starch	Attachment to epithelial and glycogen-rich alveolar cells	(63, 64)

C5a peptidase (ScpB)	C5a	Cleavage of C5a and inhibition of neutrophil recruitment to the infection site	(65, 66)

	Integrins	Adherence/invasion to epithelial cells?	(67)

	Fibronectin	Adherence to extracellular matrix	(68, 69)

Cell-surface-associated protein (CspA)	Aα subunit of fibrinogen	Cleavage of fibrinogen and increased lethality of GBS strains	(70)

	Chemokines	Inhibition of recruitment of activated neutrophils to sites of infection	(71)

### GBS Members of the CWA Proteins

#### CWA as Adhesins/Invasins and Complement Regulators

##### ACP, Alpha C Protein

Alpha C protein is the archetype of a class of surface-expressed proteins called alpha-like proteins (Alps). Members of this group of proteins are highly homologous and are supposed to share similar functions. ACP is predominantly expressed on the surface of GBS serotypes Ia, Ib, and II of GBS, while it is rarely expressed by serotype III strains (72, 73). Structurally, ACP consists of an NTD followed by a variable number of tandem repeats. The three-dimensional structure of the NTD of ACP has been solved and includes two distinct regions, named D1 and D2, respectively. D1, located at the extreme N-terminus of the domain, consists of a β sandwich, while D2 comprises three antiparallel α helix coils which in association with the adjacent repeat region constitute a binding site for heparin and glycosaminoglycans (GAGs) (36).

Functionally, the soluble form of NTD competitively blocks the internalization of GBS within human cervical epithelial cells *in vitro*, suggesting that this domain includes the major ACP determinant of cell invasion (37). Furthermore, a GBS strain expressing an ACP mutated isoform in the GAG-binding region appears to be deficient in invasion of cells, indicating that GAG-binding region mediates entry of GBS into host cells. Notably, the point mutation does not affect the rate of transcytosis of bacteria suggesting that the two processes, internalization and transcytosis, occur by distinct mechanisms and that the GAG binding function of ACP may not be necessary for transcytosis (38).

**Figure 3:**
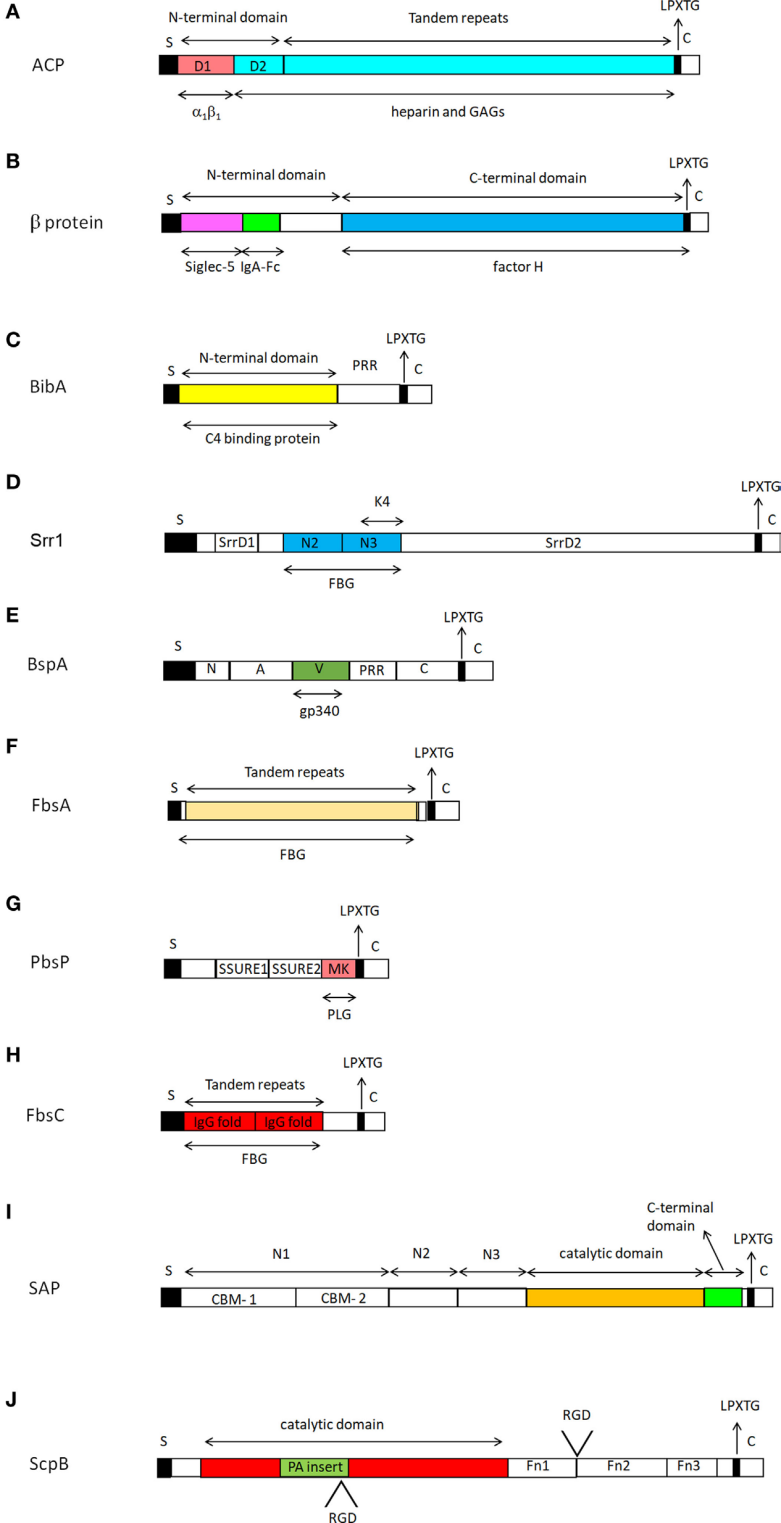
Representation of the structure of cell wall-anchored proteins from *Streptococcus agalactiae*. All cell wall-anchored *S. agalactiae* proteins contain a signal sequence (S) at the N-terminal end and a sorting signal (LPXTG) at the C-terminus. The signal sequences are removed during secretion across the cytoplasmic membrane. Each CWA protein is identified with a capital alphabetic letter **(A–J)**. Domains involved in specific ligand binding are also shown. For further details see the text. Drawings of CWA protein here reported are those for which the domain organization and ligand binding sites have been defined.

D1 shows homology to the fibronectin integrin-binding region FnIII10 and contains a KTD motif structurally similar to the classical RGD integrin-binding motif, suggesting that ACP binds α_1β1_ *via* the D1 subdomain. Conversion of KTD to KTA reduces its ability to bind to the α_1β1_ integrin and inhibits GBS internalization within ME180 cervical epithelial cells (39). Thus, there are two mechanisms that direct GBS internalization, but it remains unclear whether D1 and D2 of the NTD domain cooperate in the invasion process (Figure 3A).

Additional members of the Alp family are Rib, R28, and Alp2 proteins. The Rib protein is expressed by almost all type III strains, several type II strains and some type V strains. ACP and Rib are relatively similar but not identical in sequence both in the N-terminal and repeat domains and the two proteins do not cross-react immunologically. The R28 protein, also known as Alp3, is expressed by many *S. agalactiae* strains belonging to serotypes V and VIII. The non-repeated N-terminal region of R28 is considerably larger than the corresponding repetitive region of ACP and Rib, while the repeat region is closely homologous to that of Rib. The Alp2 protein is found only in some strains of serotypes Ia, III, and V and shows a more complex domain organization than that found in the other members of the Alp family (74, 75).

##### β Protein

The β protein, also called Bac, is a surface protein expressed by GBS strains of serotypes Ia, Ib, II, and V (74). The protein comprises two main parts, the NTDand the C-terminal domain (CTD) (76). β protein binds to the Fc moiety of human IgA (40–43) and the human complement inhibitor FH (45). The Fc-binding site has been mapped to a 73-amino acid residue region in the NTD (77). In turn, the protein binds with high affinity to the Cα2/Cα3 interdomain region in IgA-Fc and inhibits the binding of full length IgA to the human IgA receptor FcRI, which may result in the inhibition of IgA effector functions (44). The binding site for FH is distinct from that of IgA and maps to the CTD of the protein (Figure 3B) (45). β protein blocks phagocytosis by binding short consensus repeats 8–11 and 12–14 located in the middle region of FH, allowing the unbound active region to bind C3b while the C3b in the C3bFH complex is cleaved by Factor I (FI) to form iC3b (46). Human Siglec-5, a member of leukocyte cell-surface receptors, is the third ligand of β protein (47). The binding of β protein to Siglec-5 results in a series of biological effects including impairment of human leukocytes phagocytosis, oxidative burst, and extracellular trap production, generating conditions for bacterial survival in the host (48). The human Siglec-5-binding domain resides in the most N-terminal portion of the NTD (76). However, the human Siglec-5- and IgA-binding regions in the β protein NTD are well separate sites. Notably, the NTD region, but not the IgA-binding region, triggers recruitment of the tyrosine phosphatase SHP-2 to human Siglec-5 in U937 monocytic cells, an interaction which results in the inhibition of phagocytosis of GBS (Figure 3B) (76). β-protein expressing GBS also binds Siglec-14 on neutrophils through a site almost identical to that of Siglec-5. This binding serves to counterbalance the suppressive effects of β protein-mediated engagement of Siglec-5 on leukocyte activation. In response to GBS challenge, Siglec-14 promotes increased innate immune and inflammation by activating p38 mitogen-activated protein kinase and AKT signaling pathways. Thus, Siglec-5 and Siglec-14 are paired receptors that cooperate to balance immune response to pathogens (78).

##### BibA, GBS Immunogenic Bacterial Adhesion Protein

BibA is a protein containing an NTD predicted to assume a helix-rich structure followed by a proline-rich region (PRR) made of high copy number of PEAK/PDVK motifs (Figure 3C). The protein exists in four different variants and determination of sequence of *bibA* gene in a large number of isolates has shown a good correlation between BibA variants and GBS capsular serotypes (21). Expression of BibA is regulated by the CovS/CovR 2-component regulatory system, a “general” regulator of genes encoding known virulence factors such as β-hemolysins (79). Furthermore, although the majority of strains tested express BibA, surface exposure of this antigen *in vitro* was observed only in 54% of the strains examined. BibA has been found covalently associated with the bacterial cell wall and in the growth medium and both forms show an almost identical MW. It is not known whether the soluble form of BibA has a biological function. BibA is able to bind human C4-binding protein (C4BP), a modulator of the complement classical pathway, and the C4BP-binding site is located in the N-terminal region of the protein (Figure 3C). Notably, no binding was observed incubating bacteria with C4BP, suggesting that the binding site for the ligand is not available for binding on whole cells. Recombinant BibA binds to a variety of cell lines, but the cell surface receptor remains to be identified (21).

##### Srr, Serine Rich Repeat Proteins

**Figure 4:**
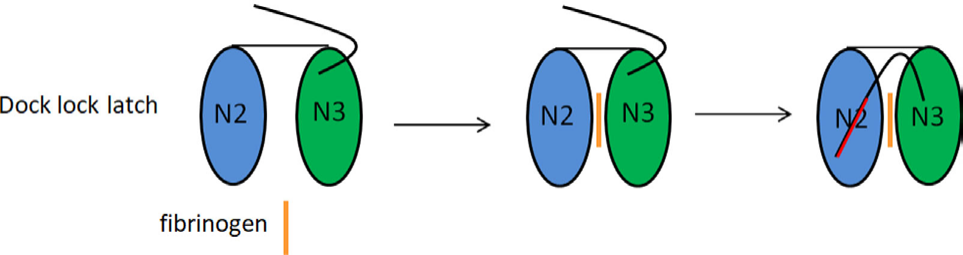
Fibrinogen binding by serine-rich repeat proteins (Srr) 1 and Srr2 by dock, lock, and latch (DLL) mechanism. The DLL mechanism is based on the observation that Srr1 and Srr2 proteins contain two subdomains, N2 and N3. The N2 and N3 subdomains concur to create a wide trench in which resides the ligand binding site. According the DLL mechanism the ligand (FBG) binding takes place in multiple steps: the ligand region first docks into the trench, followed by conformational change and redirection of the unstructured carboxy-terminal extension of the N3 subdomain, so that amino acid residues in this extension crossover the binding trench and lock the bound ligand in place. In the final latching event, the complex is further stabilized by the insertion of the N3 C-terminus through a β-strand complementation in a trench on the surface of the N2 subdomain.

The Srr proteins are a group of CWA proteins encoded by genes within loci that also encode for proteins involved in their glycosilation and export (80). The best structurally and functionally characterized Srr proteins in GBS are Srr1 and Srr2. Their organization includes a long signal sequence, a short serine-rich region, a ligand-binding region divided into the N2 and N3 subdomains, and a second lengthy serine-rich segment (Figure 3D). Glycosyltransferases attach GlcNac and sialic acid at the two serine-rich regions of Srr1. Glycosilation of Srr1 is the prerequisite for the cell surface display and for conferring resistance to proteolysis. The role of glycosylation in bacterial adherence is supported by decreased GBS binding to vaginal epithelium in the presence of N-acetylglucosamine binding lectin wheat germ agglutinin (52, 81). Further studies indicate that Srr1 interacts with cytokeratin 4 (K4) to promote bacterial attachment to vaginal cervical epithelial cells (52, 53). The binding site for K4 was localized in the non-repeat region of Srr-1 corresponding to subdomain N3 (Figure 3D), while that for Srr-1 in K4 maps to the C-terminal 255 amino acid residues (53). This finding excludes the requirement of the N2 subdomain for K4 binding and suggests a mechanism of interaction distinct from the dock, lock, and latch (DLL) mechanism of FBG binding to Srr1 (see below) (82). Both Srr1 and Srr2 bind FBG and, as measured by surface plasmon resonance spectroscopy, both interact with this ligand with different affinities. The binding site for Srr1 and Srr2 was localized to tandem repeats 6–8 (aa 283–310) of the FBG Aα chain (35). Crystal structure determination of both the Srr1 and Srr2 non-repetitive regions in combination with mutagenesis studies suggest that both the proteins interact through the combination of adjacent N2 and N3 subdomains with the segment of the FBG Aα chain *via* a DLL mechanism (35) already described for other FBG-binding adhesins, such as ClfA of *Staphylococcus aureus* (49) and SdrG of *Staphylococcus epidermidis* (Figure 4) (83). This finding is particularly relevant considering that, although Srr1 and Srr2 proteins have a limited primary sequence similarity to the above staphylococcal proteins, they have a secondary structure which resembles that of ClfA and SdrG. Srr1 binding to FBG is also an important step in the GBS colonization of tissues. In fact, attachment of GBS to human brain microvascular endothelial cells (hBMEC) *in vitro* was reduced by deletion of the putative latch segment of Srr1. Furthermore, in a mouse model of meningitis, the latch deletion was associated with significantly reduced levels of bacteria within the central nervous system (84). GBS mutant strain lacking the Srr1 latching region also exhibited decreased adherence *in vitro* and reduced persistence in a mouse model of GBS vaginal colonization, suggesting the important role of Srr1/FBG (or other potential unknown ligands) interaction in female reproductive tract colonization (50). Unlike Srr1, Srr2, which is specifically expressed by ST-17 strains of GBS, also binds to PLG which can be converted to plasmin by host PLG activators, suggesting a possible contribution in the GBS invasion of host tissues (85). Strains expressing Srr1 showed a significantly higher level of binding to human platelets as compared to isogenic *Δsrr1* deletion mutants; an interaction that was inhibited by IgG against FBG. The contribution of Srr1 to pathogenicity was further demonstrated by the higher density of a WT strain of *S. agalactiae* within heart valves, spleens and kidneys as compared to the *Δsrr1* mutant, in an endocarditis model of infection (51).

##### Leucine-Rich Repeat (LRR) Proteins

Proteins containing tandemly arranged leucine-rich repeats have been found both in prokaryotic and eukaryotic cells. In eukaryotes, LRR proteins are involved in diverse functions such as enzyme inhibition, cell adhesion, trafficking, and signal transduction (86). In prokaryotes, LRR proteins are virulence factors, the most well characterized being internalins InlA and InlB of *Listeria monocytogenes* (87). A recent study described a GBS CWA protein named leucine-rich repeat protein of GBS (LrrG) that is expressed and conserved among all serotypes of GBS. LrrG possesses an integrin-binding RGD motif that could play a role in the attachment of bacterium to host cells. The central portion of the protein consists of 15 full-length LRR sequences (each 22 amino acids long) homologous to repeats of other bacterial LRR proteins and two partial leucine-rich repeats. Recombinant LrrG protein was shown *in vitro* to bind to glutaraldehyde-fixed Hep-2 cells, suggesting that it may have a role as attachment factor (54). Thus, although the precise role of LrrG needs to be further investigated, this preliminary analysis suggests that LrrG may be a GBS virulence determinant.

##### BspA, Group B Streptococcus Surface Protein A

*In silico* analyses revealed the presence of GBS genes encoding proteins designated group B Streptococcus Surface Proteins (Bsp). At least four similar Bsp proteins (BspA, BspB, BspC, and BspD) are distributed among GBS of different serotypes (55). These proteins consist of seven distinct regions. The N-terminal half comprises a signal sequence followed by an NTD and a region A. The C-terminal half consists of a PRR followed by a CTD (C). A central variable region (V) inserted between the A and PRR regions exhibits the greatest strain-to-strain sequence variation and is supposed to confer ligand-binding specificity to each isoform (Figure 3E). The precise function of the PRR sequence in BspA (and other proteins such as BibA) is not known yet. On the basis of surface exposure of this region and of the knowledge that PRRs usually participate in protein–protein interaction it has been suggested that PRR could promote interactions with host proteins or other bacterial surface components (88). PRR could also act as a flexible stalk that allows surface exposure of other domains of CWA proteins. Finally, by analogy with proline-rich peptides such as arasin 1 (89), a proline-arginine-rich antimicrobial peptide isolated from the spider crab, one can speculate that proteolytic processing of CWA proteins could release proline-rich peptides with anti-microbial activity in the environment. Crystal structure analysis revealed that the BspA-V domain adopts a fold distinct from those reported for other Bsp family proteins and comprises a specific pocket that constitutes a potential binding site for carbohydrates or glycosylated ligands (55). BspA may have a potential role as attachment factor. In fact, expression of BspA on the surface of the surrogate *Lactococcus lactis* confers binding to Gp340, a mucin-like scavenger receptor containing numerous *N*- and O-linked glycosylation sites. Of note, BspA binding to Gp340 occurs *via* a mechanism that involves the BspA-V domain (Figure 3E). Since *L. lactis* expressing BspA also interacts with human vaginal epithelium and because these cells express high levels of Gp340, it has been suggested that BspA and perhaps other Bsp proteins, could facilitate GBS colonization of infection-relevant sites (55).

##### FbsA, Fibrinogen-Binding Surface Protein A

FbsA is a surface FBG-binding protein characterized by tandemly repeated units, each 16 amino acids in length (Figure 3F). The gene for FbsA was detected in almost all serotypes (10). The number of repeats (from 3 to 30) varies from strain to strain and *ΔfbsA* mutants show a dramatic reduction of GBS adherence to FBG, suggesting that FbSA is a GBS important FGB receptor. Western blot experiments performed with FbsA truncates demonstrated that the repeat region of FbsA contains the binding site for ligand. Competition experiments performed with synthetic peptides mimicking the repeat sequences also showed that even a single repeat unit of FbsA binds FBG. Moreover, experiments performed with synthetic peptides differing from each other by a single amino acid substitution made it possible to define a FBG-binding motif with the consensus sequence G-N/S/T-V-L-A/E/M/Q-R-R-X-K/R/W-A/D/E/N/Q-A/F/I/L/V/Y-X-X-K/R-X-X (10). The finding that growth of the *fbsA* deletion mutant in human blood is significantly impaired indicates that FbsA plays a protective role against bacterial opsonophagocytosis (10). Heterologous expression of *fbsA* in *L. lactis* promoted adhesion to but not invasion of hBMEC (57) and lung epithelial cells (A549) (58), suggesting that FbsA is a streptococcal adhesin. Consistent with the repetitive nature of the protein, FbsA was found to contain multiple binding sites for FBG. Moreover, evidence has been provided that incubation of FbsA with FBG induces extensive FBG aggregation and affects thrombin-catalyzed fibrin clot formation. FBG aggregates observed by scanning electron microscopy consisted of thick fibers organized in a cage-like structure (90). *S agalactiae* clinical isolates bearing FbsA elicit an FBG-dependent, specific aggregation of platelets, suggesting that FbsA may play an important role in thrombus formation and *S. agalactiae*-induced endocarditis (56). Notably, Siauw et al. showed that septic GBS strains bind FBG, induce thromboxane synthesis and platelet aggregation, while colonizing GBS strains cause only shape change of platelets (91). Liu et al. also found that strains of GBS isolated from septic arthritis can induce platelet aggregation and activation *via* the elevation of platelet Toll-like receptor 2 expression (92). However, no information is provided on the possible involvement of specific CWA proteins in this important process by these studies. Hence, so far FbsA remains the only identified GBS surface protein playing a role in platelet aggregation/activation.

##### PbsP, Plasminogen-Binding Surface Protein

Recently in a capsular serotype III strain belonging to the clonal complex 23 a gene was identified that encodes a putative CWA protein termed PbsP. This protein consists of two ~150-aa streptococcal surface repeat (SSURE) domains displaying 77% identity and a methionine/lysine-rich (MK-rich) region (Figure 3G) (93). PbsP is expressed by almost all clinical GBS isolates and shows a 99% identity among the strains (94). PbsP mediates PLG binding both *in vitro* and *in vivo*. The main PLG-binding motif is localized in the MK-rich region of PbsP (20), whereas the functional role of the SSURE domains remains to be elucidated. PLG captured on the surface of GBS expressing PbsP can be activated into plasmin by tissue PLG activators and this results in an increase of bacterial extracellular proteolytic activity. Conversely, a *pbsP* deletion mutant shows a decreased bacterial transmigration across brain endothelial cells and an impaired virulence in a murine model of infection (20).

##### HvgA, Hypervirulent GBS Adhesin

Late-onset disease (LOD) is a GBS-associated, deadly syndrome which can occur after the first week of life and is characterized by septicemia and a high rate of associated meningitis (95). The great majority of LOD cases are caused by a single hypervirulent clone GBS ST-17, which expresses the CWA protein HvgA as a signature virulence factor (59, 96). The 5′ and 3′ ends of the genes encoding HvgA and BibA are highly conserved, whereas their internal regions show a reduced level of sequence identity (59). GBS strains that express HvgA adhere more efficiently to several cell lines including intestinal epithelial cells and microvascular endothelial cells that constitute the BBB than do strains lacking HvgA. In mice orally inoculated with GBS expressing HvgA, the protein appears to be a critical factor in the intestinal colonization and translocation across the intestinal barrier and the BBB, indicating that HvgA is an important causative agent of meningitis (59). GBS isolated from body fluids of infected mice expresses higher HvgA levels than GBS cultured *in vitro*, indicating that HvgA expression is upregulated during infection. Furthermore, the ability of GBS ST-17 to spread from the intestine to other organs or tissues is linked to the age of the mice, since 60–70% of 2–3 weeks old mice undergo rapid death after enteric infection with *hvgA*-expressing GBS, whereas mice older than 4 weeks are protected (59). In conclusion, HvgA is a GBS virulence determinant in the neonatal context and appears a promising target for immune-based interventions. An in-depth dissection of structural/functional organization of HvgA remains to be elucidated.

##### FbsC, Fibrinogen-Binding Surface Protein C

FbsC is a surface protein encoded by the prototype GBS strain NEM316 (60). It bears two bacterial immunoglobulin-like tandem repeat domains, possibly involved in host ligand binding (Figure 3H). When compared to other FBG-binding proteins from GBS or other organisms, FbsC exhibits an almost unique sequence, although its general organization with two large internal IgG-like tandem repeat domains appears similar to other bacterial surface proteins (60). Along this line a CWA protein termed BsaB was described in GBS strain 515 which promotes GBS binding to Fn and Lm and to Fn-coated ME-180 cervical epithelial cells (62). A comparative analysis of the sequence of FbsC and BsaB revealed that the two proteins are identical and encoded by the same gene. With the exception of strains belonging to the hypervirulent lineage ST-17, FbsC is expressed by almost all GBS clinical isolates. The two-component CovRS regulatory system downregulates also expression of FbsC and other FBG-binding proteins. Consistent with this, inactivation of CovRS leads to a dramatic increase in GBS binding to FBG (60). Recombinant FbsC protein specifically binds to FBG with an affinity that is intermediate between that of FbsB (16) and FbsA (16, 97) and predominantly binds to the FBG Bβ chain (60). Moreover, FbsC is apparently essential for the ability of GBS to bind FBG, because binding is almost completely abrogated in a *ΔfbsC* mutant, a phenotype that can be reverted by a complementation with an FbsC-expressing plasmid. The finding that *ΔfbsC* mutant shows a significant reduction in GBS attachment to and invasion of lung and intestine epithelial cells and the observation that lung and intestine are the bacterial entry portals in early- and late-onset GBS disease indicates that FbsC may be an important virulence factor in neonatal GBS-promoted infection (60). In this context, because FGB accumulates on the surface of epithelial cells (94), it is plausible that FbsC-dependent adherence and invasion of epithelial cells are mediated by GBS/FBG interaction. Finally, immunization of mice with recombinant FbsC significantly protected the animals from letal GBS challenge, suggesting its potential use as a component of a multivalent anti-GBS vaccine (60).

#### CWA Proteins With Enzymatic Activities

In a similar fashion to other streptococcal species, some CWA proteins from *S. agalactiae*, as well as interacting with host ligands, exhibit specific enzymatic activities.

##### SAP, *Streptococcus agalactiae* Pullulanase

Analysis of the GBS genome sequences revealed a novel putative CWA protein, SAP which was subsequently shown to metabolize α-glucans (pullulanase) (63). The genome of the large majority of GBS isolates harbors the *sap* gene and homologous genes have been described in other pathogenic streptococci (64, 98). SAP is a large mosaic protein containing five highly conserved domains: the N1 domain including two putative carbohydrate-binding motifs (CBMs), a pullulanase-associated domain (N2 domain), an isoamylase-like domain (N3 domain), a glycoside hydrolase domain with a catalytic triad comprising Asp, Glu, and Asp and a C-terminal β-sandwich domain (C domain). Of note, the second CBM also contains a fibronectin type III repeat-like region (Figure 3I) (63). A crystal structure of the catalytic domain of SAP, both in its apo form and in complex with maltotetraose (MTT), a four saccharide-cleavage product of glycogen, has been determined. The three-dimensional structures of apo and MTT-bound SAP are quite similar, suggesting that binding of synthetic substrate does not induce conformational changes in the enzyme. The substrate binding to the catalytic domain is principally stabilized by hydrogen bonds and aromatic-sugar interactions between MTT and SAP. Ca^2+^ and Cl^−^ ions were also found in the crystal structure of both the apo and MTT-bound SAP. It has been suggested that the Ca^2+^ ions stabilize the interaction between the N2 and N3 domains and that the Cl^−^ anions are likely involved in creating an electrostatic environment for substrate binding (99). SAP is able to degrade α-glucan polysaccharides, such as pullulan, a linear polysaccharide of maltotriosyl repeating units linked by α-(1,6) or other homopolysaccarides such as glycogen and starch. α-Glucan polysaccharides present in the growth medium induced an up-expression of SAP on bacterial surface. Furthermore, deletion of the *sap* gene resulted in a reduction in bacterial growth in a medium containing pullulan or glycogen. Consistent with this, incubation of GBS with immune sera against SAP reduced the capacity of the bacterium to degrade pullulan (63). Together these findings confirm the importance of SAP in GBS metabolism of α-glycans and envisage its possible use as potential vaccine component.

##### ScpB, Streptococcal C5a Peptidase B

Streptococcal C5a peptidase B, also known as C5a peptidase, is a 120 kDa GBS surface protease and adhesin/invasin expressed by all GBS serotypes. ScpB is a multidomain protein composed of an N-terminal subtilisin-like protease domain, an insertion termed the protease-associated (PA) domain within the protease domain and three Fn type III domains (Fn1-Fn3) at the C-terminal end (Figure 3J). Protease activity of this CWA protein is dependent on the catalytic triad formed by Asp, His and Ser which form the putative active site of the enzyme. ScpB specifically cleaves and inactivates human C5a by releasing a seven-residue C-terminal segment, abolishing the binding capability of C5a for the C5a receptor on neutrophils and the consequent recruitment of neutrophils to the site of infection (65, 66). Conversely, the enzyme does not inactivate C5a from other animal species including the mouse and does not block the chemiotactic recruitment of neutrophils promoted by mouse C5a (66, 100). It has been questioned whether the C5a-cleaving activity is the main role of ScpB because there are strains belonging to the highly virulent III-3 clone of GBS that express an ScpB protein lacking enzyme activity (101). Two Arg-Gly-Asp (RGD) sequences located in the PA domain and between Fn1 and Fn2, respectively, interact with host integrins. Conversion of either aspartate to alanine partially abrogates binding to epithelial cells, while substitution of aspartate with alanine in both RGD motifs completely eliminates binding of ScpB to the cells (67). The discovery that rabbit anti-ScpB serum inhibits invasion of epithelial cells indicates that ScpB is essential for colonization of epithelium by GBS (102), whereas it is unclear whether the RGD motifs mediate adherence to and invasion of host cells by GBS. Purified ScpB protein also binds with high affinity to solid phase-attached, but not soluble Fn. This preferential binding to insoluble Fn could be due to the formation of conformational determinants produced by the juxtaposition of several Fn molecules on a solid surface (68, 69). Naturally occurring variants of ScpB with a deletion that abolishes C5 peptidase function maintain the ability to interact with Fn, suggesting that the Fn-binding activity of ScpB resides in a region which is independent of its enzymatic activity (68, 103).

##### CspA, Cell-Surface-Associated Protein A

Group B *Streptococcus* expresses another surface-associated serine protease named CspA which is under control of the MtaR transcription factor (104). Clinical isolates from all capsular serotypes harbor the *cspA* gene (70). Sequence analysis indicates that CspA is a subtilisin-like serine protease, with strong homology to ScpB and caseinases from lactobacillales. CspA is a multidomain protein containing an N-terminal PRO (propeptide) domain, a serine protease domain and a domain A of unknown function. After synthesis and anchoring to the cell wall, CspA removes its N-terminal PRO domain autocatalytically and, eventually, cleaves its own C-terminus, resulting in release from the cell surface (71). Unlike other CWA proteins which display polar (PilB) or uniform distribution (Alp2), CspA shows a preferential punctate localization on the bacterial surface (105). Studies by Harris et al. have shown that the highly virulent type III GBS isolate COH1 cleaves the Aα subunit of FBG at a specific site, whereas a *cspA* mutant is unable to do so. Moreover, incubation of FBG with *S. agalactiae* cells results in the formation of fibrin-like aggregates. A *cspA*-mutant was much less virulent in a neonatal rat sepsis model and is more prone than the wild type strain to be phagocytosed by human neutrophils *in vitro*, suggesting that cleavage of FBG by CspA may increase the virulence of GBS, possibly increasing the bacterial protection from opsonophagocytic attack (70). Finally, CspA possesses the ability to inactivate the main representative CXC chemokines (GRO-α, GRO-β, GROγ, neutrophil-activating peptides 2, and granulocyte chemotactic protein 2) and this prevents activated neutrophils from migrating to sites of infection (71).

## Immunological Properties of CWA Proteins and Their Potential as Vaccine Candidates

A central issue in vaccine development lies in the selection of bacterial antigens. In general, antigens for a vaccine are selected because (i) they are highly immunogenic; (ii) they are located on the bacterial cell surface where they will be more accessible to the IgG; (iii) they are expressed at high levels *in vitro* and *in vivo*; (iv) they have a wide distribution by the majority strains and low sequence variability; (v) they play a role as virulence factors; and (vi) antibodies against a CWA protein are able to prevent the biological function of the antigen and facilitate clearance of the organism through phagocytosis. Several CWA proteins meet the criteria listed above and are potential antigens that can be used as vaccine to combat GBS infections. Indeed, a number of experiments have been performed to test the value of CWA proteins individually or in combination as vaccine. Rib confers protection to animals lethally infected with strains expressing the protein (106). Purified or recombinant ACP elicits antibodies that protect mice against infection (72). Therefore, a vaccine composed of Rib and ACP might confer a broad serotype-independent protection (107). ScpB, a protein almost universally expressed by GBS strains and with little or no antigenic variability, elicited protective antibodies and induced serotype-independent protection (108). BibA induced opsonizing antibodies and protected mice challenged with a strain expressing high levels of the protein, suggesting a potential role of this antigen as a vaccine candidate (109). A protection level induced by vaccination with FbsA or BibA in a model of lethal sepsis in mice was variable and dependent on the challenge strain. Notably, vaccination with FbsA and BibA showed itself to be more effective with respect to individual antigens. Consistent with this, FbsA and BibA hyperimmune, specific antibodies generated a high and broad level of protection in mice challenged with GBS strains in passive immunization studies (110). In addition, maternal immunization with an FbsA antigen consisting of five repetitive units significantly protected pups against lethal GBS challenge. Furthermore, a monoclonal antibody that neutralized FBG binding by FbsA saved pups against lethal GBS challenge (97). Immunization of mice with different Srr1 truncated peptides demonstrated that only animals vaccinated with Srr1 truncates containing the latch sequence were protected from GBS meningitis. Moreover, the latch peptide alone induced protective antibodies against different GBS serogroups, suggesting the potential use of the latch peptide as a vaccine component (111). Finally, LrrG protein, although poorly exposed on the surface of *S. agalactiae* grown *in vitro*, when tested as a vaccine antigen, was immunogenic and elicited protection against experimental infection with GBS, suggesting that it is exposed on the bacterial surface during growth *in vivo* (54). Summing up, several CWA proteins showed themselves to be effective in both passive and active immunization experiments. On the contrary, although *in vitro* studies suggest a possibile function in pathogenesis for the above proteins, conclusive evidence that CWA proteins contribute to GBS diseases is still lacking. In fact, it is unclear whether proteins such as LrrG, BspA, CspA, and SAP are virulence factors and consequently involved in colonization, evasion and/or inhibition of the host’s immune response and entry and exit out of the host cells. Neither is any information available on the role of strains expressing these proteins and their deletion mutants in animal models of infection. Finally, the presence of CWA proteins such as Srr2, ACP, β protein, and HvgA in a minority of GBS serotypes limits their potential use in a global GBS vaccine.

## Conclusion and Perspectives

In this review, we have updated the role played in host colonization and infection by several surface proteins from GBS discovered in recent years. Many of these proteins are adhesins that interact with FBG or Fn and some of them are involved in the internalization into host cells. It is amazing how little is known about interference by CWA proteins on the activities of innate immunity. In view of the fact that the latter strategy is commonly used by other bacterial species, notably *Streptococcus pyogenes* (112), further work is needed in this direction. We also need to understand the regulation of expression of these factors in different growth phases especially by clinically important strains. High-resolution crystal structure of CWA proteins in the apo-form and in complex with the appropriate ligand may allow the design of novel GBS therapeutic agents. Studies based on the atomic resolution of CWA protein structure could also provide important information for the definition of new critical immunological epitopes. Conserved protein antigens expressed on the surface of bacteria represent crucial elements for the formulation of new therapeutic tools (vaccines) against GBS. In the recent past unconjugated CPSs have been successfully used for the development of a vaccine against GBS (113), although they elicit independent B cell activation on T-lymphocytes and do not induce B-cell memory response. Conversely, polysaccarides conjugated with a protein carrier have the potential to efficiently induce T-lymphocyte dependent immunity. For example, the use of *S. agalactiae* β protein as the carrier of the type III polysaccaride elicited antibodies against both components and resulted in protective immunity against infection of *S. agalactiae* expressing either component (114). To overcome limitations in the use of capsular vaccines, more recently efforts have also been directed toward the identification of novel protein vaccine candidates, among these pilus subunits. Again, due to the antigenic variation associated with the pilin subunits, full coverage against all GBS strains was not possible (115–117). Thus, perspectively, the combination of well conserved CWA determinants with pilus subunits or CPSs could make an important contribution to the development or improvement of a multivalent, effective anti-GBS vaccine.

## Author Contributions

PS, GP, SR, CA, and LM wrote the manuscript. GP and SR prepared the references and figures. PS finalized the manuscript.

## Conflict of Interest Statement

The authors declare that the research was conducted in the absence of any commercial or financial relationships that could be construed as a potential conflict of interest.
